# Deep learning features encode interpretable morphologies within histological images

**DOI:** 10.1038/s41598-022-13541-2

**Published:** 2022-06-08

**Authors:** Ali Foroughi pour, Brian S. White, Jonghanne Park, Todd B. Sheridan, Jeffrey H. Chuang

**Affiliations:** 1 The Jackson Laboratory for Genomic Medicine, 10 Discovery Dr., Farmington, CT 06032 USA; 2 Department of Pathology, Hartford hospital, 80 Seymour St, Hartford, CT 06106 USA; 3 Department of Genetics and Genome Sciences, UCONN Health, Farmington, CT 06032 USA

**Keywords:** Computational biology and bioinformatics, Computational models, Image processing, Machine learning, Statistical methods

## Abstract

Convolutional neural networks (CNNs) are revolutionizing digital pathology by enabling machine learning-based classification of a variety of phenotypes from hematoxylin and eosin (H&E) whole slide images (WSIs), but the interpretation of CNNs remains difficult. Most studies have considered interpretability in a post hoc fashion, e.g. by presenting example regions with strongly predicted class labels. However, such an approach does not explain the biological features that contribute to correct predictions. To address this problem, here we investigate the interpretability of H&E-derived CNN features (the feature weights in the final layer of a transfer-learning-based architecture). While many studies have incorporated CNN features into predictive models, there has been little empirical study of their properties. We show such features can be construed as abstract morphological genes (“mones”) with strong independent associations to biological phenotypes. Many mones are specific to individual cancer types, while others are found in multiple cancers especially from related tissue types. We also observe that mone-mone correlations are strong and robustly preserved across related cancers. Importantly, linear mone-based classifiers can very accurately separate 38 distinct classes (19 tumor types and their adjacent normals, AUC = $97.1\% \pm 2.8\%$ for each class prediction), and linear classifiers are also highly effective for universal tumor detection (AUC = $99.2\% \pm 0.12\%$). This linearity provides evidence that individual mones or correlated mone clusters may be associated with interpretable histopathological features or other patient characteristics. In particular, the statistical similarity of mones to gene expression values allows integrative mone analysis via expression-based bioinformatics approaches. We observe strong correlations between individual mones and individual gene expression values, notably mones associated with collagen gene expression in ovarian cancer. Mone-expression comparisons also indicate that immunoglobulin expression can be identified using mones in colon adenocarcinoma and that immune activity can be identified across multiple cancer types, and we verify these findings by expert histopathological review. Our work demonstrates that mones provide a morphological H&E decomposition that can be effectively associated with diverse phenotypes, analogous to the interpretability of transcription via gene expression values. Our work also demonstrates mones can be interpreted without using a classifier as a proxy.

## Introduction

Deep learning has become an important methodology for analyzing biomedical images, and in particular for analyzing hematoxylin and eosin (H&E) stained whole slide images (WSIs). Deep neural networks have achieved classification accuracies higher than classical machine learning models^1^. However, they are black-boxes that do not directly reveal the morphological features they associate with labels, a significant concern for mechanistic analysis and clinical decision making^2^. Identification of biologically meaningful morphological features may be confounded by image artifacts^3^, such as blurring, noise, and lossy image compression^4^. Tissue damage, image quality, and dataset-specific artifacts have also been suggested to affect feature representation and prediction accuracy of neural networks^1,5,6^. Given the impact of such artifacts on deep learning-based predictors, it is of critical importance to be able to decompose CNNs into features that can be biologically interpreted.

The majority of models for visualizing, analyzing, and interpreting CNNs reveal “where” a network is “looking” to make its prediction, rather than revealing “what” information in the region of interest is important. Some methods output pixel patterns that affect the value of a neuron in a deep network^7^. However, such techniques tend to output different predictive regions, can be difficult to validate, or have been suggested to be “fragile”, i.e. extremely sensitive to small perturbations of the image^8^. Optimizing conventional deep learning techniques, such as self-attention, to identify regions informative of class labels is a current theme in digital pathology^9,10^. While most methods assess deep feature representations as a whole, recent work suggests deep learning features cluster together and encode distinct morphologies^11^. Other recent works have focused on visualizing individual deep learning features as heatmaps^12^. Finally, as the majority of interpretation methods have focused on identifying regions predictive of class labels, they requires a trained classifier and cannot be directly used in pipelines that employ unsupervised feature learning.

Unlike natural image analysis^13^, biomedical image analysis is complemented by additional data modalities, such as multiplexed imaging, single cell and bulk sequencing, and clinical information^14,15^. These data may aid in interpreting the deep feature representations of the H&E slide. However, models integrating these diverse modalities are needed. The feasibility of doing so is supported by work establishing the connection between modalities, for example by using CNNs to predict expression values of specific genes from H&E images^16–18^. Because of the architectural complexity of CNNs, it has often been assumed that CNN-based decompositions of images into features are not interpretable. However, there has been little empirical study of this question, e.g. by testing whether CNN-derived features are correlated with simple biological features such as gene expression values.

In this work, we investigate the interpretability of CNN-derived image features. Prior works^1,19^ have referred to these by various names (e.g. features, fingerprints ) whose use is not specific to biological image analysis. For clarity and because they represent morphological features in many ways analogous to genes, we refer to them as mones (i.e. “morphological genes”). We find that mones share statistical similarities with gene expression data, and hence, a mone can be conceptualized as an abstract gene with some expression value. Individual mones have strong linear associations with phenotypic features, making them directly interpretable, which we demonstrate in several analyses. We demonstrate that many mones can distinguish cancer tumors from adjacent normal slides. These mones can be linearly combined for reliable prediction of both pan-cancer and tissue-specific cancer phenotypes. Mone-mone correlation analysis identifies clusters of highly correlated mones within cancer types, and these correlations are strongly preserved among cancers from related tissues. The similarity of mone values to gene expression data allows immediate use of many interpretable bioinformatics tools and machine learning models to identify the underlying biology of morphologies encoded by CNNs. For example, integrative mone-gene expression correlation analysis reveals that collagen content and immune infiltration are linearly associated with morphologies encoded by mones in several cancer types, and we confirm these relationships by expert histopathological review. Our studies confirm individual deep learning features encode distinct and identifiable morphology, and demonstrate the power of mones for computationally deconstructing cancer images into interpretable biological features.The linear analysis of individual deep learning features versus expression values or interpretable morphology provides a simple and effective approach to interpreting deep learning models in biological image analysis, notably without the need for a trained classifier.

## Results

**Figure 1:**
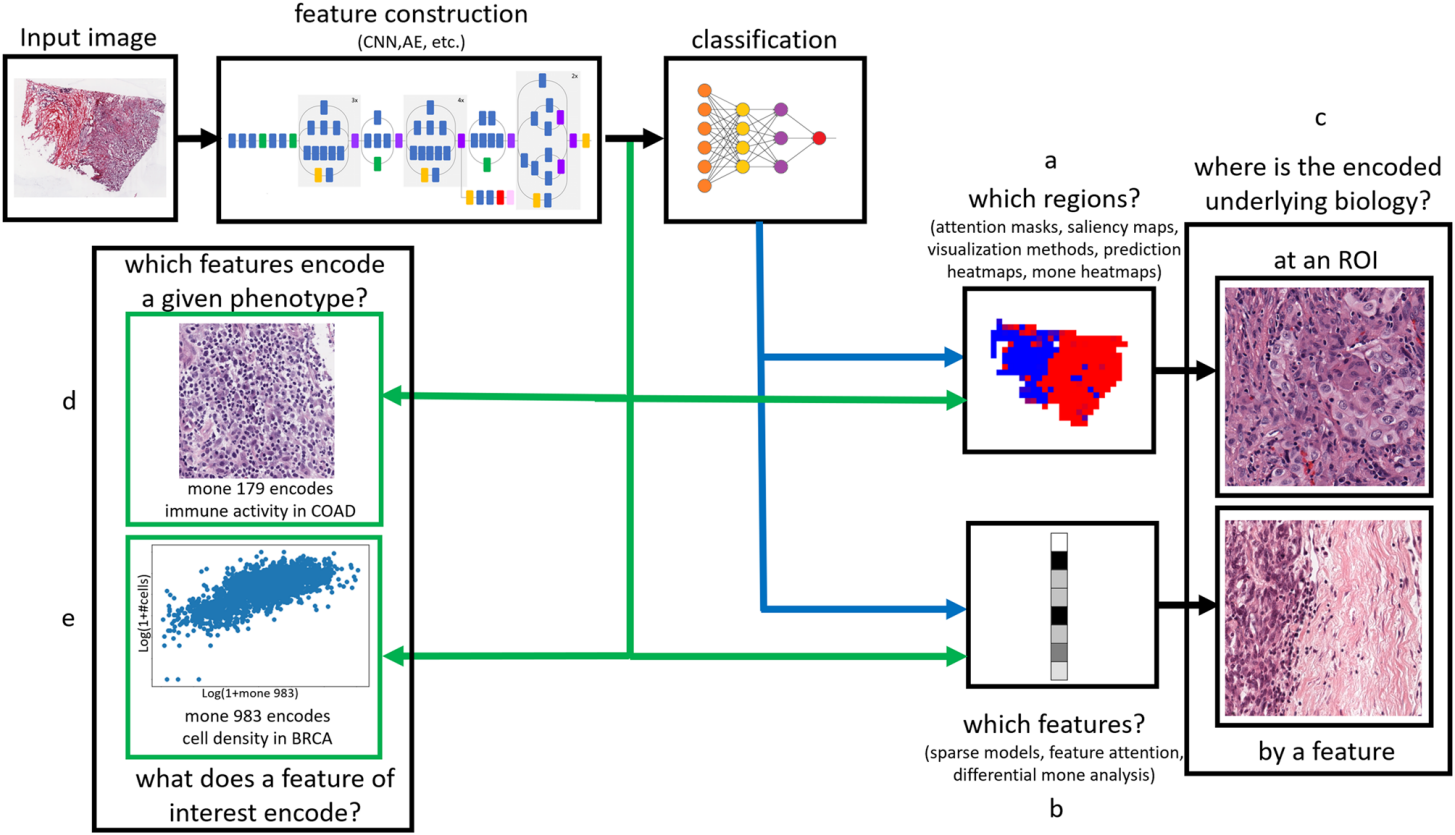
An overview of interpretation methods in deep learning. Blue arrows denote methods that require a trained classifier, and green arrows denote methods that do not require a trained classifier. (**a**) Several methods identify regions which drive the network’s prediction. These masks can be generated by the network, e.g. spatial self-attention^62^, or as a post-process via visualization methods^7^ such as GradCam, or prediction heatmaps^1^. Heatmaps of individual mones and mone-based classifiers can be used to detect predictive regions. (**b**) Channel attention^62^ and sparse models, including sparse mone-based classifiers, identify subsets of features that are predictive of class labels. Differential mone analysis identifies discriminative features without training of a classifier. (**c**) Methods in (**a**) and (**b**) are typically used to select example image regions that have high attention, affect predictions the most, or affect the value of a feature. Mone analysis can be used to (**d**) identify features that encode a given phenotype of interest and (**e**) identify the morphology a feature of interest encodes without training a classification model.

We analyzed the InceptionV3^20^ features of tiles from whole slide images of The Cancer Genome Atlas (TCGA)^1^ for 19 cancers (see Supplementary File 1 for the full list). We used features derived from this architecture because predictive models based on Inception have shown high accuracy for identifying phenotypes in prior studies^1^. We hereafter denote each of the 2048 outputs of the global average pooling layer of the Inception V3 network as mones (morphological genes). We use this terminology because mones have analogies to genes with individual expression values. Tile level mones can be combined to construct slide level mones (see “Methods”). Unless otherwise stated, in the studies below “mone” refers to a slide level characterization. Figure 1 provides an overview of interpretive mone analyses and their connection to current interpretation techniques in the field.

### Individual mones differentiate phenotypes

**Figure 2:**
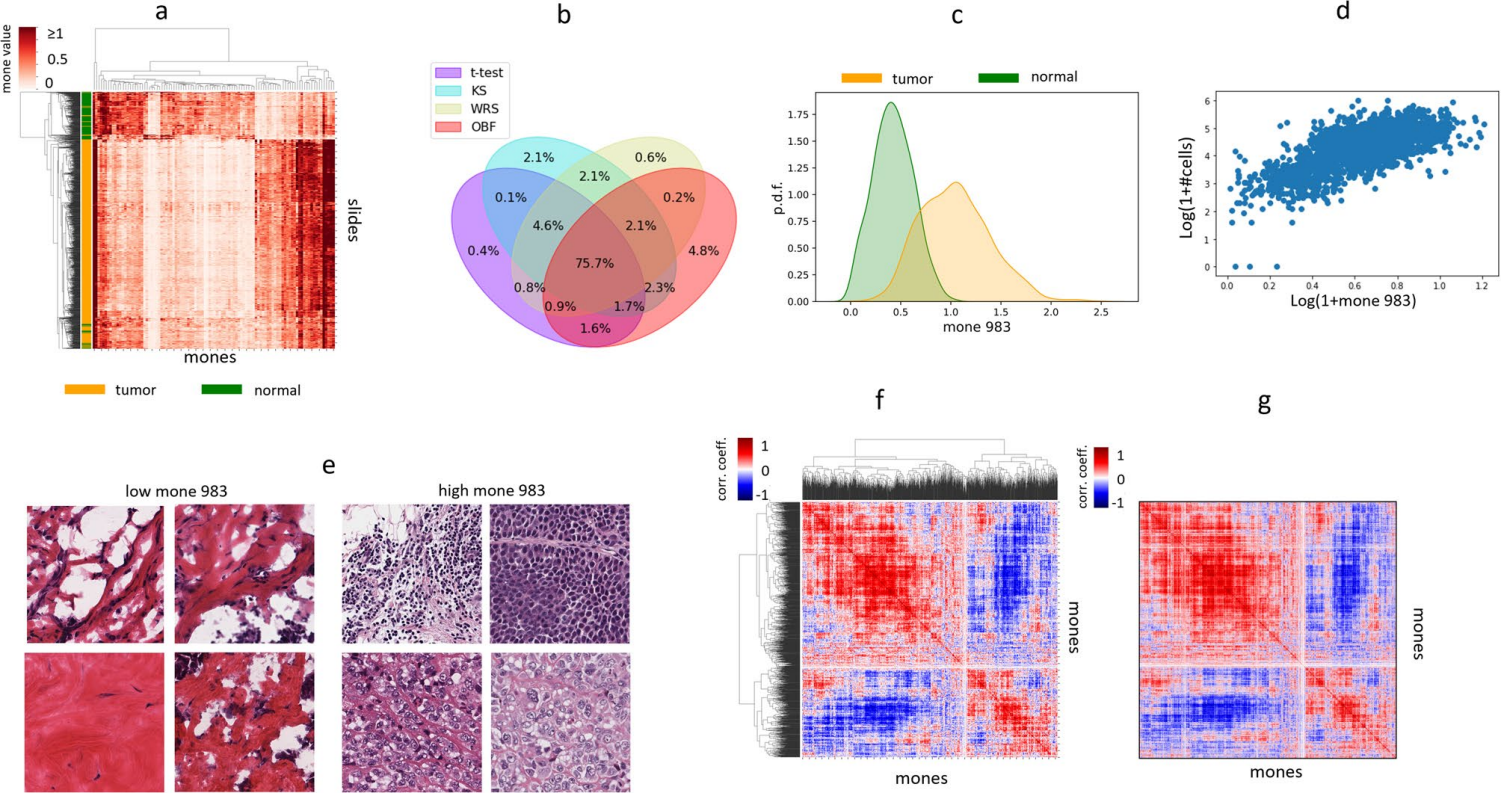
Individual mones and mone pairs encode and distinguish phenotypes. (**a**) Clustermap of BRCA slides using the top100 mones differentiating the slides. 100 mones are sufficient to separate frozen normal (green) from frozen tumor (orange) slides. (**b**) Venn diagram of statistically significant mones differentiating tumor from adjacent normal frozen slides, comparing different statistical tests. Venn diagrams were calculated for each cancer type, and the observed plot shows the average across all cancer types. On average the statistical tests agree on $75\%$ of mones differentiating between tumor and normal slides. (**c**) Probability density function of mone 983 among frozen tumor (orange) and adjacent normal (green) BRCA slides. (**d**) Log-normalized scatter plot of slide level mone 983 and cellpose^57^ estimates of cellularity across BRCA frozen slides. (**e**) Example tiles from slides with extreme mone 983 values (high and low). (**f**) Cluster map of the mone-mone correlation matrix of LUAD tumor slides, demonstrating that many mones pairs are highly correlated. (**g**) Mone-mone correlation matrix of LUSC slides, with mones ordered identically to the Fig. 1f cluster map.

We first investigated to what extent individual mones can differentiate phenotypes, focusing on TCGA tumor/normal slide comparisons. We initially identified individual mones with significant differences in distribution between breast cancer (BRCA) tumor and adjacent normal slides (> 1800 mones were statistically significant with FDR < 5%). A clustermap of the top 100 such mones was able to clearly separate these classes (see “Methods”, Fig. 2a, and Supplementary Fig. 1, clustermap AUC = 89%, rand score = 96%, and adjusted rand score = 85%). These results were typical of mone behaviors in many tumor types—in any given tumor type, many mones were able to separate frozen tumor from normal slides (see “Methods” and Supplementary File 1). We applied several statistical methods to test the robustness of such mones. In each cancer, at least $40\%$ of mones were statistically significant irrespective of the statistical test used (FDR = 5%, Supplementary File 1), and $75.7\%$ of mones significant by at least one method were significant by all tests (see “Methods”, Fig. 2b). A smaller subset of these mones showed strong effect sizes, as identified by optimal Bayesian filter (OBF)^21^ statistics (see “Methods”). $22\%$ of all mone-cancer pairs met this criterion based on distributional differences between tumor and normal slides (Supplementary Fig. 2).

As an example, mone 983 is a tumor marker with a distributional difference between frozen tumor and adjacent normal breast cancer (BRCA) slides (Fig. 2c). It also behaves similarly in several other tumor types (Supplementary Fig. 3). It strongly correlates with cell density in frozen BRCA slides (see “Methods”, Fig. 2d,e, Pearson r = 0.69, p-value < 1e−200, Supplementary File 2) and significantly though with moderate magnitude in FFPE BRCA tumor slides (Pearson r = 0.24, p-value = 1.9e−13). Mone 983 has higher correlation with cell density in FFPE LUAD slides (Pearson r = 0.61, p-value = 2.2e−34) than frozen LUAD slides (Pearson r = 0.28, p-value = 4.1e−18, Supplementary File 2).

To further test whether mones exhibit consistent behavior in different cancer types, we analyzed the four cancer families of^1^: pan-GYN, pan-KIDNEY, pan-LUNG, and pan-GI (Supplementary File 3 for cancers in each family). More than half of the mones with distributional differences in each cancer type also have distributional differences in all cancers of the family (Supplementary File 3). Although cancer-specific mones are uncommon, such mones still show clear distributional differences between tumor and normal (Supplementary Fig. 4). Interestingly, most mones distinguishing tumor from normal in both LUAD and LUSC also distinguish between the LUAD and LUSC cancers (Supplementary File 3), suggesting quantitative values are important. For example, mone 1914 distinguishes LUAD and LUSC frozen (adjusted t-test p-value < 1e−50) and FFPE (adjusted t-test p-value < 2.1e−28) tumor slides, where LUAD slides tend to have a higher mone1914 value (Supplementary Fig. 5).

Individual mones can also distinguish frozen from FFPE slides (Supplementary File 4). We observed the ability of some mones to distinguish between tumor and normal is impacted by differences between frozen and FFPE modalities ($421 \pm 112$ across all cancers, see “Methods”, Supplementary File 4), though the majority of mones behave similarly in frozen and FFPE.

### Mone clusters provide robust encodings of cancer phenotypes

We next investigated to what extent mones are independent or encode behaviors together. We did this by calculating pairs of mones that were significantly correlated, for each cancer type. We analyzed this first by restricting to frozen slides (to avoid Simpson’s paradox), and second by combining frozen and FFPE slides (to avoid false correlations due to frozen-specific artifacts). The results were generally robust between the two methods-a few cancers had a non-trivial difference in the ratio of correlated mone pairs ascertained by the two methods (ESCA, KICH, SARC, and PRAD $22.3\% \pm 2.22\%$), while the remaining cancers had small differences ($7.8\% \pm 3.9\%$, Supplementary File 6). Therefore we subsequently analyzed frozen samples only unless otherwise specified. Overall, we found that correlated mones are prevalent among tumor slides (Fig. 2f,g, and Supplementary File 6). For example, $83.9\%$ and $88.7\%$ of mone-mone pairs are correlated within LUAD and within LUSC, respectively (see “Methods” and Supplementary File 6). We observed similar results for other cancers: $68.8\% \pm 13\%$ of all mone-pairs were statistically significant across cancers (Supplementary File 6). Remarkably, we observed that pairwise correlations are preserved within cancer families—more than $45\%$ of mone-pairs statistically significant in one cancer are significant in all cancers of the family (pan-GYN, pan-KIDNEY, pan-LUNG, and pan-GI, Supplementary Fig. 6). For example, the mone-mone correlations in lung adenocarcinoma and lung squamous cell carcinoma are nearly identical (Fig. 2f,g). We also calculated mone-mone correlations in the normal slides associated with each cancer type, hypothesizing that the difference in mone-mone correlations between tumor and normal might be important to distinguishing tumor from normal images. However, these differential mone correlations are weaker and less preserved across cancers (Supplementary File 6 and Supplementary Fig. 6).

Different cancers can be distinguished by different sets of mones. For example, while mone 983 separates tumor and normal slides of BRCA, it does not differentiate COAD tumor and normal slides (Supplementary Fig. 3). We identified 105 mones that are highly correlated with mone 983 in BRCA (Null: $|r| \le 0.5$, FDR = $0.1\%$, see “Methods”), among which 52, 14, and 29 also differentiate tumor from normal slides in COAD, READ, and STAD, respectively (t-test FDR < $0.1\%$). The first principal component of these COAD-overlapping mones (explaining $41\%$ of the variance across COAD samples) strongly correlates with cell density in frozen slides (Cellpose cellularity estimates: Pearson r = 0.22, p-value = 2.2e−11, HoverNet cellularity estimates: Pearson r = 0.43, P-value = 1.9e−47, see “Methods” and Supplementary File 2). Thus the high cellularity in BRCA^22,23^ and COAD^24,25^ involve incompletely overlapping mone sets.

### Linear models of mones can detect and distinguish tumors

**Figure 3:**
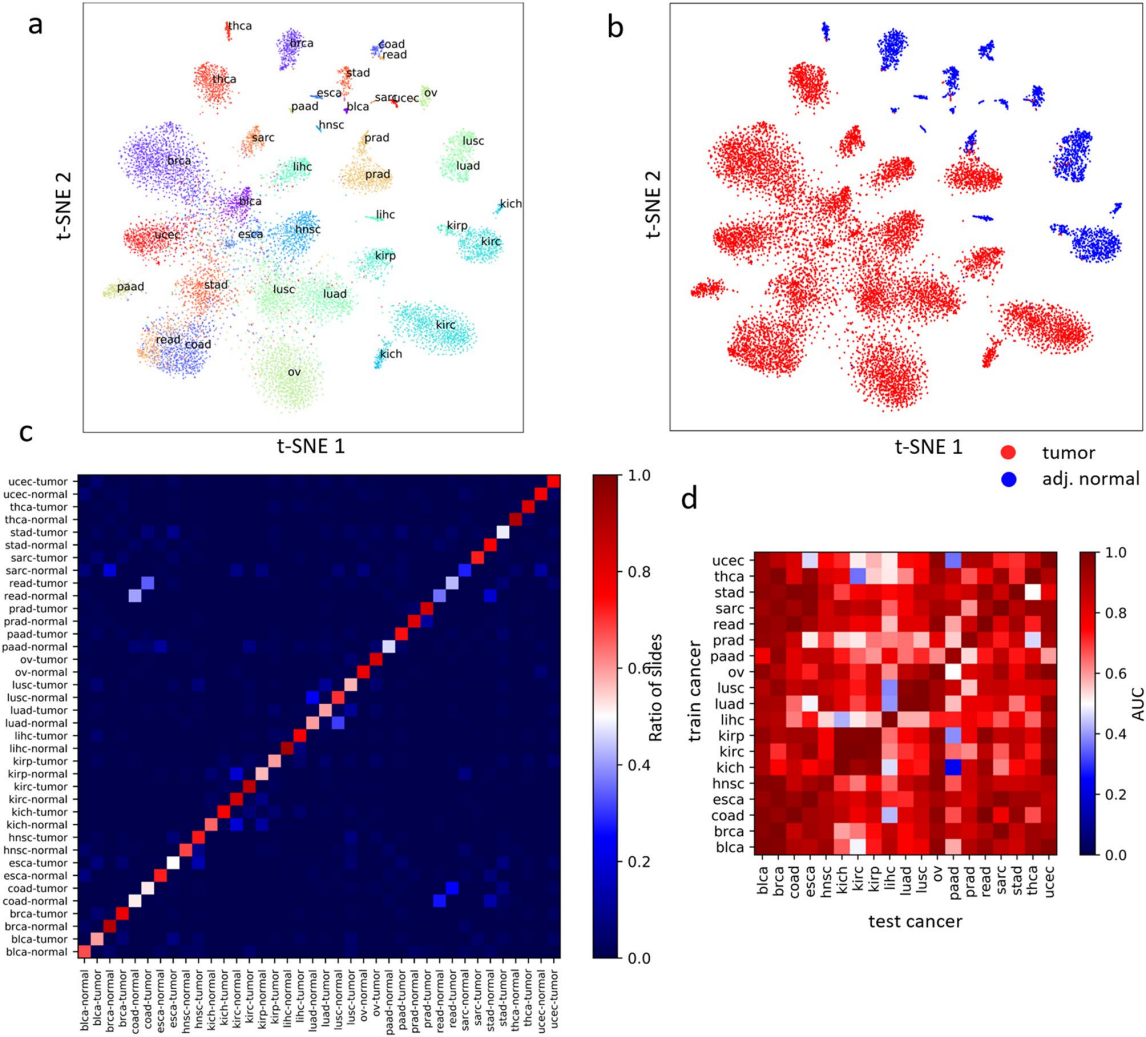
The joint distribution of mones reliably separates tumor and normal slides and the underlying cancer. 2D t-SNE plots of the mone-based MLDA feature space distinguishing 38 classes (19 cancers, tumor/normal status) based on (**a**) cancer type and (**b**) tumor/normal status. (**c**) Normalized confusion matrix of the 38-class mone-based logistic regression classifier. The color depicts the ratio of slides with a given true class predicted as any of the possible classes. The large diagonal values suggest the classifier has high accuracy. (**d**) The cross-classification AUCs of mone-based logistic regression tumor/normal classifier trained on each cancer and applied to all cancers.

We investigated linear models of mones for predicting phenotypes, as they allow direct interpretation of mone values. We observed that linear models of mones can efficiently distinguish tumor from adjacent normal slides, as well as the cancer type from which they are derived (19 cancers, 38 classes, see “Methods”, Fig. 3a–c). We tested two linear models, multi-class linear discriminant analysis [MLDA, One versus Rest (OVR)-AUC = $97.1\%\pm 4.6\%$, Supplementary File 7] and multinomial logistic regression with LASSO penalty (LR-LASSO, OVR-AUC = $97.1\% \pm 4.2\%$, Supplementary File 7). MLDA encodes mone patterns indicative of class labels into a low dimensional space (i.e. the number of classes - 1), yielding t-SNE visualizations with improved interpretability over naïve t-SNE (compare Fig. 3a,b and Supplementary Fig. 7a,b). LR-LASSO, on the other hand, is a linear model based on small mone sets, so its regression coefficients can be interpreted directly with risks incurred by each mone.

Although CNN methods typically use difficult-to-interpret fully connected layers at the classification step, we found that efficiently designed linear models can replace fully connected layers while still achieving high prediction AUCs. Combining tumor probabilities of the LR-LASSO classifier we obtained a universal tumor detector with extremely high AUC ($99.2\% \pm 0.12\%$, see “Methods”), out-performing the fully deep learning model of^26^ (Reported AUC = $0.95 \pm 0.02$). Furthermore, LR-LASSO is effective at cross-classification similar to CNNs with fully connected classification layers^1^, i.e., LR-LASSO trained to distinguish tumor/normal for one cancer type can distinguish tumor/normal for other cancer types as well (Fig. 3d and Supplementary Fig. 7c). While the LR-LASSO model has smaller average AUC (0.84) compared to the fully deep learning model of^1^ (0.88), logistic regression is more interpretable than a multi-layer perceptron. Our slide level tumor detectors also produce meaningful tile level predictions. Independent review by our pathology team supports most tumor regions having high tumor probability, and most non-tumor regions have low tumor probability in these images, with the cases of misclassification tending to be prediction of non-tumor regions to be tumor (Supplementary Fig. 8). These results indicate that the LR-LASSO slide level tumor markers are effective at the tile level.

### Mones have interpretable correlations with gene expression

We next investigated whether mones are linearly associated with gene expression values, as this could provide transcriptional interpretability for mones (see “Methods” for data stratification and pre-processing). We observed many mones significantly correlated with individual genes. Across five cancers analyzed (OV, COAD, KIRC, LUAD, and LUSC) between 83 (LUSC) and 1797 (KIRC) mones were associated with at least one gene, whereas between 332 (LUSC) and 16474 (KIRC) genes were associated with at least one mone (“Methods”, Supplementary File 8). We then analyzed several cases of particular interest.

#### Mones encode collagen content

**Figure 4:**
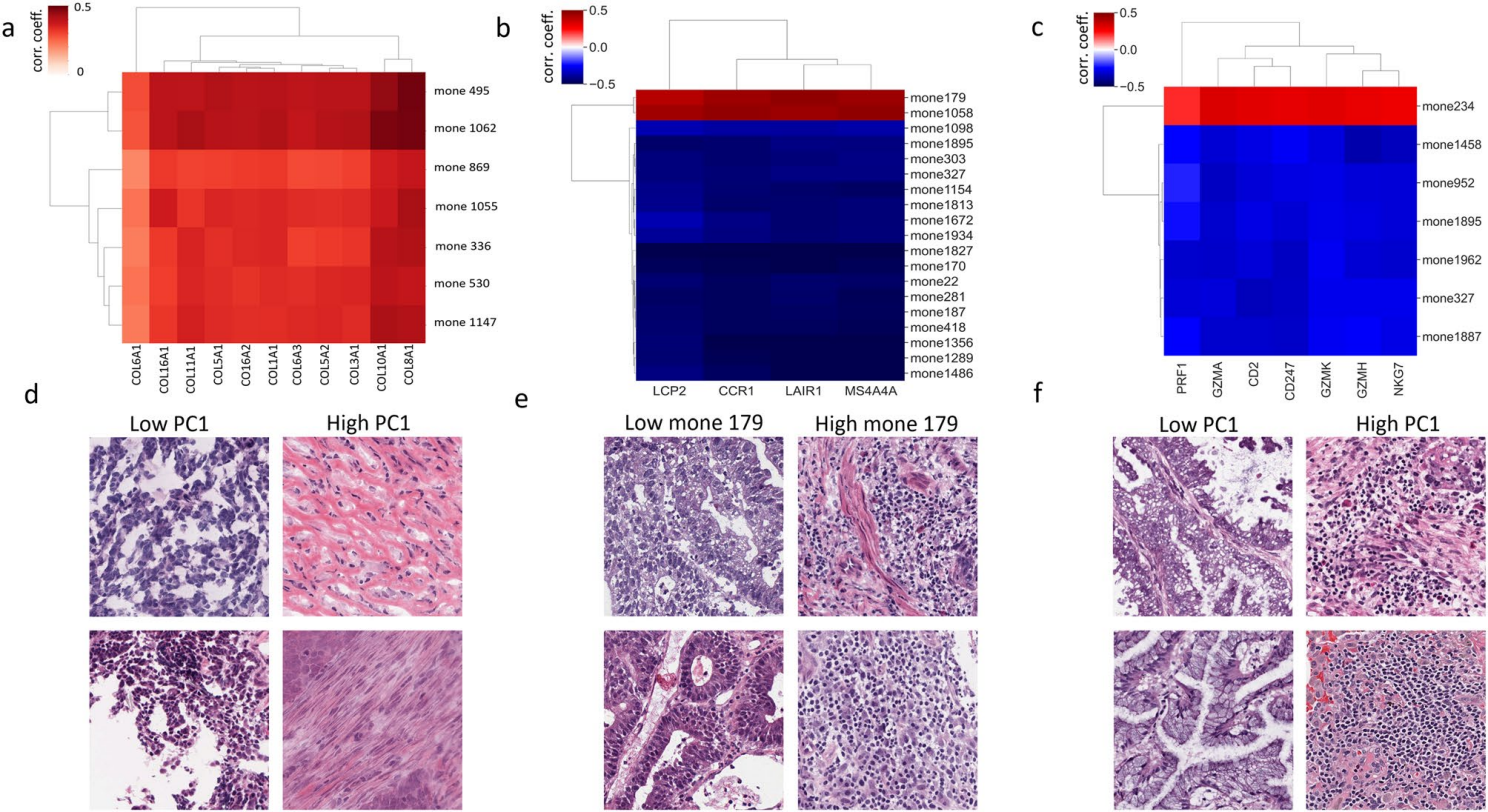
Mone-gene correlation analysis identifies highly correlated mone-gene clusters. Correlation matrix of (**a**) a cluster of highly correlated mones and collagen genes in OV , and a cluster of highly correlated mones and immune-related genes in (**b**) pan-GI cancers and (**c**) LUAD. See Supplementary Fig. 9 for adjusted p-values. Example tiles from slides with (**d**) high and low PC-1 in OV, (**e**) high and low mone 179 in COAD, and (**f**) high and low PC-1 in LUAD. Histopathology review identifies that mone-predicted (**d**) OV tiles with high PC-1 are rich in collagen, and (**e**) COAD tiles with high mone 179 and (**f**) luad tiles with high PC-1 have a strong lymphocyte presence. See Supplementary Figs. 10–13 for additional examples at both high and low mone values.

We first used unsupervised analysis to study clusters of correlated mone-gene pairs. We identified a cluster of highly correlated mones and collagen genes in OV (Fig. 4a and Supplementary Fig. 9). These mone values can be efficiently combined for association with phenotype using PCA (PC-1 explains $63\%$ of the variance). Histopathological review by our pathology team confirmed tiles with high PC-1 values as typically rich in collagen (Fig. 4d and Supplementary Fig. 10), and tiles with low PC-1 values as having low collagen but increased cellularity. These mone-gene associations may be clinically relevant, as high expression of collagen genes correlates with multi-drug resistance^27^ and poor prognosis in ovarian cancers^28^. Mone 1062—one of the mones in the identified cluster—was additionally highly correlated with ECM2, THBS1 and THBS2, which have been suggested to play a significant role in ovarian cancer drug resistance and metastasis^28–30^.

#### Mones encode immune infiltration

Supervised correlation analysis using fixed gene sets can also be used to test if mones encode morphological features associated with a biological phenotype. We tested whether mones encode immune infiltration in pan-GI (COAD, READ, and STAD) and pan-LUNG (LUAD and LUSC) cancers, with immune related gene sets taken from^31^ and^32^ (see “Methods” and Supplementary File 9).

We identified 19 mones significantly correlating (some positive, some negative) with immune related genes in pan-GI cancers (Fig. 4b and Supplementary File 9). These mones have significant correlations with each other (|r| = $0.56 \pm 0.13$), and 14 mones significantly correlate with more than one immune gene. LAIR1, LCP2, MS4A4A, and CCR1 correlate with 15, 9, 12, and 15 mones, respectively. All 19 mones are significantly correlated with prior TCGA estimates of leukocyte fraction^33^ (FDR $<0.05,|r|=0.29 \pm 0.07$) and HoverNet estimates of immune cell quantity from the H&E images (see “Methods”, FDR $<0.05, |r|=0.45 \pm 0.12$) in COAD. Mone 179 had strong positive correlations with both leukocyte fraction (r = 0.2) and HoverNet estimates (r = 0.65). Histopathological review confirmed that mone 179 differentiates amongst COAD tiles according to their level of immune infiltration (Fig. 4e and Supplementary Fig. 11). PC-1 of the 19 mones has significant correlation with leukocyte fraction (r = 0.4, p-value = 2.4e−35) and HoverNet estimates (r = 0.17, p-value = 2.5e−17). Histopathological review validated that PC-1 also differentiates COAD tiles according to differing levels of immune infiltration (Supplementary Fig. 12). Thus PC-1 efficiently combines mones and provides a stronger separation than individual mones.

We observed similar correlations, but smaller in magnitude, between mones and local immune cytolytic activity genes in lung cancers (Fig. 3c and Supplementary File 10). We observed stronger correlations in LUAD than LUSC (LUAD |r| = $0.20 \pm 0.05$, LUSC |r| = $0.12 \pm 0.05$). We identified 31 mones significantly correlating (some positive, some negative) with immune related genes in LUAD. 7 mones correlated with at least 3 genes (Fig. 4c). Only one LUAD immune mone did not correlate with lymphocyte fraction (FDR = 0.05). PC-1 of the 31 mones correlates with lymphocyte fraction (r = 0.28, p-value = 2.2e−4). Histopathological review of LUAD slides based on PC-1 suggests LUAD tiles with high PC-1 show a strong tumor infiltrating lymphocyte presence and have inflammation, while tiles with low PC-1 are typically not inflamed and show weaker immune infiltration (Fig. 4f, Supplementary Fig. 13).

#### Mones identify immunoglobulin gene expression in highly cellular colon adenocarcinoma tumors

Supervised correlation analysis can also clarify finer behaviors within WSIs. For example, highly cellular COAD tumors typically show high expression of immunoglobulin (IG) genes. We considered the 52 mones correlated with mone 983 and which differentiate COAD tumor from normal (“Mone clusters provide robust encodings of cancer phenotypes” section). The PC-1 dimension of these mones significantly correlates with 87 genes in COAD (FDR $<0.05$, Supplementary File 11 for the full gene list), as well with the average expression of these 87 genes (r = 0.33, p-value = 3.8e−12). Immunoglobulin (IG) genes dominate this gene set, and we define their average expression as a sample’s IG score.

B-cells express immunoglobulin, and as expected we observed a statistically significant correlation between the log normalized B-cell estimates of^33^ and IG score (p-value = 2.4e−12). Interestingly, however, B cells comprise only a small fraction of cells in each sample ($0.86\%\pm 1.5\%$), and we did not observe a significant correlation between the B-cell estimates and mone PC-1 (correlation coefficient = − 0.009, P-value = 0.85). Thus mone analysis suggests that IG expression is not due solely to B-cells. This supports recent studies suggesting colon cancer cells themselves express IG genes^34^.

### External validation on CPTAC

We externally validated mone patterns on CPTAC-LUAD^35^ and CPTAC-LUSC^36^. Mone 983 correlates with cell density in CPTAC-LUSC (r = 0.41, p-value = 1.1e−40, Supplementary Fig. 14) and CPTAC-LUAD (r = 0.21, p-value = 1.8e−12, Supplementary Fig. 14). Pathologist evaluation confirmed the ability of the PC-1 dimension of TCGA-LUAD immune mones (“Mones encode immune infiltration” section) to separate CPTAC-LUAD slides by immune activity. We also observed that HoverNet frequently mislabels lymphocytes as dead cells (Supplementary Fig. 14). Therefore, mone analysis has more robust behavior on CPTAC-LUAD than HoverNet (Supplementary Fig. 14).

Similar to TCGA, CPTAC-LUAD slides tend to have a higher mone 1914 value than LUSC slides (t-test raw p-value < 1e−50). Pathologist review suggests LUAD slides with high values of mone 1914 tend to have gland and micropapillary formations, and have finely vacuolated cytoplasm (Supplementary Fig. 5). These features are indicative of adenocarcinoma and were not observed in CPTAC-LUSC slides with low mone 1914 values (Supplementary Fig. 5).

## Discussion

Mone analysis provides interpretability of deep neural networks through the linear correlations of deep learning features against phenotypes and gene expression profiles. While the richness of CNN features suggests potential linear correlations of individual features with human interpretable morphologies, validation of such relations is necessary, as human interpretability is not explicitly enforced in the training of CNNs. Furthermore, identification of the encoded morphology by each mone is a non-trivial task. Although correlation is not causation, our mone analysis empirically shows that mones efficiently encode strong morphological features that can often be used to replace multi-layer perceptrons with robust and interpretable linear classification models. Mone-analysis is flexible, can be used in a diverse set of interpretation modalities, and can be applied to features engineered through various training methodologies. Moreover, we have demonstrated that integrative mone-gene correlation analysis can identify specific transcriptional processes from images, and verified these through expert pathological review.

### Mones provide image-based interpretability

Linear models based on mones have several empirical and theoretical strengths for image analysis. Individual mones and small mone clusters directly correlate with phenotypes (“Individual mones differentiate phenotypes” and “Linear models of mones can detect and distinguish tumors” sections), enabling a simpler interpretation of CNNs compared with methods that integrate all CNN features together. Most interpretation models assume deep feature representations are complex and non-linear, and therefore provide interpretability primarily through example regions identified by problem-specific classifiers. On the other hand, our results demonstrate CNNs decompose H&E stained images into interpretable features that linearly correlate with phenotypes, and the highly non-linear feature representation assumption can be relaxed for interpreting CNNs trained on H&E slides.

Some prior linear analyses of deep learning features have enabled partial interpretation of CNNs trained on one cancer type^12,37^. In contrast to^12^, which only reported on individual mones through their heatmaps overlaid with H&E images, our mone analysis identifies individually correlated mones and genes, providing finer interpretability. The canonical correlation analysis of^37^ also has less interpretability than our approach as it investigates many-to-many relationships between mones and gene expression. These works also focus on individual cancers, while our study provides pan-cancer interpretations. For example, our results demonstrate a pan-cancer mone can encode conserved morphological features across multiple cancer types (“Individual mones differentiate phenotypes” section), and a conserved morphological feature can be encoded by distinct mones in different cancer types (“Mone clusters provide robust encodings of cancer phenotypes” section). Moreover, mones within strongly correlated clusters can be linearly combined to better identify shared encoded morphology (“Mone clusters provide robust encodings of cancer phenotypes” section and^11^).

Pan-cancer mones and correlations between mones can facilitate and improve robustness of interpretation across cancer types. For example, mone983 encodes cellularity (both in TCGA and CPTAC, see “Individual mones differentiate phenotypes” and “External validation on CPTAC” sections) and distinguishes tumor and normal slides of multiple cancers (Supplementary Fig. 3), but not GI cancers (Supplementary Fig. 3). However, mones encoding cellularity in COAD can be identified through their correlations with mone983 (“Mone clusters provide robust encodings of cancer phenotypes” section), even though mone983 is not itself associated with cellularity in COAD. Another example is correlation of mone1895 and mone327 with immune genes in LUAD and pan-GI cancers (Fig. 4).

Mone analysis improves low dimensional visualization of large image sets, such as via t-SNE plots. Pre-trained CNNs are universal feature extractors that encode morphological features predictive of a multitude of labels^1,38,39^. Because not all high-dimensional complex relations can be easily embedded in 2D, mone analysis can be used as a first phase of targeted search to identify the mones relevant to classes of interest (“Linear models of mones can detect and distinguish tumors” section), allowing more accurate visualization of class separations in mone space.

Mone analysis guides classifier design by exploring statistical properties of the learned features. For example, linear associations of correlated mone clusters with phenotypes suggests utility of sparse linear models for reliable classification (“Linear models of mones can detect and distinguish tumors” and “External validation on CPTAC” sections). Robustness of linear models using a small number of mones (“Linear models of mones can detect and distinguish tumors” and “External validation on CPTAC” sections) provides empirical evidence for the theoretical results establishing robustness of sparse deep learning models^40–42^. Furthermore, removing mones encoding tissue-specific structures and keeping mones encoding morphological features of interest can help build classifiers robust to tissue specific patterns.

Correlation analysis of mones with gene expression values is a powerful approach for interpreting mones. We identified clusters of highly correlated mone-gene sets, demonstrating clear connection of mones to the underlying genetics. Some recent studies have used exhaustive sets of deep learning features to predict expression profiles^16–18^, but our work shows that small mone-gene clusters can be sufficient and provide simpler interpretability. Both supervised and unsupervised analyses identify meaningful clusters (“Mones have interpretable correlations with gene expression” section). Supervised analysis using fixed gene sets is particularly interesting, as it enables direct assessment of genes of interest via mones (“Mones encode immune infiltration” section).

Strong correlations of individual mones with image-derived features (“Individual mones differentiate phenotypes” section and Fig. 1d) or gene expression values (“Mones have interpretable correlations with gene expression” section and Fig. 4b) facilitate direct interpretation, but moderate correlations require further analysis. Dimensionality reduction methods, such as PCA, combine moderately correlated mones and boost the associations between mones and the encoded morphology. Whether moderate correlations are due to multiple mones being weak detectors of a complex morphology, individual mones partially encoding multiple morphologies, or gene expression profiles having moderate correlation with morphology (see^16–18^ for examples) are open research questions.

### Interpretation without classifier training

A notable advantage of the mone approach is that it does not require a trained classifier, which is especially desirable when feature engineering and classification are decoupled (e.g. transfer learning, unsupervised learning). Interpretation models that require a trained classifier, such as attention-based models, are restricted to the morphologies that are predictive of a predetermined set of classes and are utilized by the classifier. Additionally, different classification architectures applied to a fixed learned feature space may use different features and morphologies for predicting a class label. Mone analysis does not require a classifier to determine if the learned features encode a given phenotype (“Mones encode immune infiltration” and “Mones identify immunoglobulin gene expression in highly cellular colon adenocarcinoma tumors” sections). It can be immediately applied to any learned feature space irrespective of training methodology, and can be used in an unsupervised fashion to identify encoded morphologies (“Mones encode collagen content” section).

Optimization of training classifiers that are robust to stain differences across datasets is an open research question. However, an advantage of mone analysis is that it can be more robust to stain differences than pure classification models, as we observed for cell classification, where our mone-based model enjoyed better generalization to external datasets than HoverNet (“External validation on CPTAC” section). It can also be used to analyze how stain differences affect feature representations and a classifier’s ability to make reliable predictions.

### Mone analysis across architectures and data modalities

Our analyses demonstrate that mones provide an efficient and interpretable CNN embedding of image data, but a caveat is that they have been restricted to Inception V3 mones. Architectures with fully connected layers tend to increase non-linearity in feature representations. Therefore, models that do not utilize multiple sequences of fully connected layers, such as Inception, are more appropriate for linear mone analysis. For example, recent work suggests a small subset of VGG19 features may also be interpreted via their direct association with phenotypes^12^. However, we believe Inception V3 mones are more appropriate for linear association studies because they are the direct inputs to the classification layer. A few other studies have explored correlations between deep autoencoder features and gene^37^ or protein expression^43^ for other architectures, but the relations between mones across architectures remains a broad and open research topic. We have found that Inception V3 mone 983 in BRCA can be reliably estimated via linear models using ResNet152V3 and DenseNet201 mones ($R^2>0.95$). Furthermore, we have been able to convert Xception mones to Inception V3 mones using autoencoders with reasonable accuracy ($R^2 \approx 0.5$). Recent work suggests InceptionV3 and ResNet features are almost equivalent^44^.These studies suggest that integrative linear mone-gene correlation analysis can be made effective across a range of deep learning architectures.

While this work focuses solely on H&E WSIs, we believe mone-based interpretation will be valuable for extension to other spatial data types. Immunohistochemistry (IHC) images are primed for rapid progress, as recent work has shown that several IHC markers can be virtualized from H&E^45,46^. Generalizing mone analysis for other data types such as spatial transcriptomics and multi-channel protein data is also an exciting and open area, though new architectures will need to be explored to handle the high dimensionality of such images. The interpretation of CNNs for these image types is a challenging but important task, and we expect that integrative multi-modal multi-architecture mone analysis will be a potent and informative approach.

## Methods

### Data acquisition and pre-processing

The 20X H&E WSIs of TCGA were pre-processed, tiled, and passed through an InceptionV3 model pre-trained on image-net data as described in^1^. Consistent with^1^ non-overlapping 512 pixel-by-512 pixel tiles were used, and only tiles containing at least $50\%$ tissue were passed through InceptionV3. The cached 2048 global average pooling layer features of InceptionV3 (called mones in this manuscript) were written to disk and analyzed. For each of these 2048 mones, the median value across all tiles within a slide was computed to yield the slide-level mone value. CPTAC-LUAD and CPTAC-LUSC cohorts were processed similarly.

### Differential mone analysis

Differential mone analysis identifies mones with statistically significant distributional differences across classes. Welch’s t-test, Kolmogorov Smirnov (KS) test, Wilcoxon Rank Sum (WRS) test, and optimal Bayesian Filter (OBF) (see^21^ for details) were used for statistical analysis. t-test, WRS test, and KS test use the Benjamini-Hochberg procedure^47^ for FDR correction. The scipy python package^48^ was used to implement t-test, KS test, and WRS test. The statsmodels^49^ implementation of the Benjamini-Hochberg procedure was used.

Minimal risk OBF (see^21^ for details) identifies mones with posterior probabilities larger than $1-\alpha$, where $\alpha$ is the FDR rate. FDR-OBF (see^50^ for details) outputs the feature set that bounds the sample conditioned FDR by $\alpha$. OBF can report the FDR of any arbitrary feature set. Unless otherwise stated FDR-OBF is used. OBF uses Jeffrey’s prior, assumes the prior probability of a mone having distributional differences is $50\%$ (to model no preference on the identity of a mone, i.e., with or without distributional differences across classes), and sets the normalization constant of the prior to 0.1. Mones with distributional differences across classes are hereafter called markers, and mones without distributional differences are called non-markers. The posterior probabilities of OBF can be used to estimate the first two moments (mean and standard deviation) of the number of markers (see^50^ for details). Assuming mone identities (marker or non-marker) are independent across cancers, the posterior probabilities can be multiplied to calculate the probability of a joint event.

OBF intrinsically computes the ratio between sample variance and weighted geometric mean of class-conditioned variances, hereafter denoted by *a*(*m*) for mone *m*. Similar to many ANOVA-based analyses this ratio measures distributional differences and is closely related to Bhattacharyya distance^51^. It converges to 1 for non-marker mones and converges to larger values for markers. Larger *a*(*m*) values denote larger distributional differences. Assuming balanced samples of sizes 200 and 100 we compute the *a*(*m*) values resulting in a posterior of 0.95 as thresholds to distinguish moderate [a(m) = 1.088] and strong [a(m) = 1.159] mones separating tumor from normal slides (Supplementary Fig. 2).

Structured multi-class OBF (see^52^ for details) considers the four possible relations (known as structures by OBF) between frozen normal, frozen tumor, and FFPE tumor slides: (A) a mone does not differentiate between slides (prior probability = 0.5), (B) a mone has one distribution for frozen slides (both tumor and adjacent normal slides) and another distribution for FFPE slides (prior probability = 0.5/3), (C) a mone has one distribution for tumor slides (both frozen and FFPE) and another distribution for frozen normal slides (prior probability = 0.5/3), and (D) a mone has one distribution for FFPE tumor slides and frozen adjacent normal slides and another distribution for frozen tumor slides. Mones with structure B for which frozen tumor and FFPE tumors lie on both sides of frozen adjacent normal slides (based on mean values) are considered ineffective due to FFPE/frozen differences.

### Mone correlation analysis

For each cancer type, we calculated correlations between mones over all samples of the given cancer type. This analysis was done for each cancer type. We use the Ledoit-Wolf shrinkage^53^ implementation of the scikit-learn python package^54^ for computing covariance matrices. We then compute the correlation matrix from the covariance matrix. We apply the Fisher transform to correlation coefficients and approximate the null with its asymptotic Gaussian distribution. Benjamini-Hochberg^47^ procedure is used for FDR correction. We use seaborn package^55^ with default values to generate clustergrams. Correlation matrices are averaged to compute the pooled correlation matrix. Statistically significant mone-mone correlations are referred to as “correlated mone pairs”.

Differential mone correlations denote the difference between the correlation coefficient of tumor and normal slides, i.e., for each cancer, differential matrix is computed by subtracting the correlation coefficient matrix of normal slides form the correlation coefficient matrix of tumor slides. Differential mone correlation analysis uses the asymptotic Gaussian distribution of the difference between Fisher transformed correlation coefficients to compute the p-values. Statistically significant differential mone correlations are referred to as “differentially correlated mone pairs”.

### Linear classification models

We implement MLDA and LR-LASSO using the scikit-learn python package^54^ with default values except we set $C=100$ and use the “saga” solver^56^ in non-binary problems for LR-LASSO. We observed little sensitivity of AUCs to the *C* values ranging from 1 to 1000, and hence use $C=100$ throughout. We adopted a Monte-Carlo cross-validation strategy and randomly split data to train and test sets 10 times using scikit-learn’s train$\_$test$\_$split function. The splits were made at the patient level, and class ratios were preserved across train and test portions. The mean and variance of the AUCs across all splits are reported. We used the Scipy package^48^ to implement the Wilcoxon signed rank test to compare AUCs.

2D t-SNE visualizations of the MLDA space were implemented using scikit-learn. PCA initialization was used to improve reproducibility of the t-SNE plots and separation of classes. Number of neighbors was set to 50. All other parameters were set to the default values.

### Cell segmentation and classification

Cellpose^57^ was used to segment and count number of cells in BRCA, LUAD, and COAD tiles. Given the fixed magnification and tile size the number of cells per tile captures tile level cell density. Median number of cells per tile was used as slide level cell density index. HoverNet^58^ was used to segment, count, and classify nuclei within COAD tumor slides. HoverNet was executed using the pre-trained PanNuke model^59^, such that nuclei were classified into one of five types: neoplastic epithelial, non-neoplastic epithelial, connective (including fibroblasts and endothelial), inflammatory (including leukocytes, lymphocytes, and macrophages), and dead nuclei. Median number of cells nuclei across tiles were used as cell density. Median number of predicted inflammatory nuclei across tiles were used to characterize presence of immune cells.

#### Integrative mone-gene analysis

Gene expression data were downloaded from the GDC portal^60^. We only used slide-gene expression pairs where both the slide and the expression profile were from the same vial. Log normalized FPKMs were used. Genes with zero counts in more than half the mone-gene pairs or expression standard deviation below 0.25 were removed. Given a set of mone-gene pairs, we stack the mone and gene vectors and compute the covariance matrix using the Ledoit-Wolf shrinkage method^53^ implemented in the scikit-learn python package^54^. Correlation values are computed given the covariance matrix similar to mone correlation analyses. Statistical significance tests are performed similar to mone correlation analyses.

#### Immune profiling and analysis

Leukocyte fractions of TCGA samples were obtained from^33^. All T-cell and B-cell categories were summed to obtain T-cell and B-cell proportions, respectively. The fractions of T-cell and B-cells were summed to obtain lymphocyte fractions. Log normalization of fractions were used throughout. Correlation analysis of immune scores with mones and IG score was performed similar to mone correlation analysis. B-cell percentages above $3\%$ were removed for computing the B-cell correlations as they were deemed outliers.

## Supplementary Information

Supplementary Figures.

Supplementary Information.

## Data Availability

TCGA data are publicly available through the GDC portal (https://portal.gdc.cancer.gov) and CPTAC data are publicly available through the cancer imaging archive^61^ portal (https://www.cancerimagingarchive.net/).

## Code availability

The code for mone analysis is available on github (https://github.com/aforoughipour/mone).

## References

[1] Noorbakhsh J, Farahmand S, Namburi S, Caruana D, Rimm D, Soltanieh-ha M, Zarringhalam K, Chuang JH (2020). Deep learning-based cross-classifications reveal conserved spatial behaviors within tumor histological images. Nat. Commun..

[2] Miotto R, Wang F, Wang S, Jiang X, Dudley JT (2018). Deep learning for healthcare: Review, opportunities and challenges. Brief. Bioinform..

[3] Wang S, Yang DM, Rong R, Zhan X, Fujimoto J, Liu H, Minna J, Wistuba II, Xie Y, Xiao G (2019). Artificial intelligence in lung cancer pathology image analysis. Cancers.

[4] Dodge, S. & Karam L. Understanding how image quality affects deep neural networks. In *2016 Eighth International Conference on Quality of Multimedia Experience (QoMEX)*, 1–6. (IEEE, 2016).

[5] Nair, T., Foroughi pour A. & Chuang, J. H. The effect of blurring on lung cancer subtype classification accuracy of convolutional neural networks. In *IEEE Conference on Bioinformatics and Biomedicine*, 2987–2989 (IEEE, 2020).

[6] Howard, F. M. *et al.* The impact of digital histopathology batch effect on deep learning model accuracy and bias. *bioRxiv* (2020).

[7] Alber M, Lapuschkin S, Seegerer P, Hägele M, Schütt KT, Montavon G, Samek W, Müller KR, Dähne S, Kindermans PJ (2019). iNNvestigate neural networks!. J. Mach. Learn. Res..

[8] Ghorbani, A., Abid, A. & Zou, J. Interpretation of neural networks is fragile. In *Proceedings of the AAAI Conference on Artificial Intelligence* Vol. 33, 3681–3688 (2019).

[9] Lu, M. Y. *et al.* Deep learning-based computational pathology predicts origins for cancers of unknown primary. arXiv preprint arXiv:2006.13932 (2020).

[10] Hägele M, Seegerer P, Lapuschkin S, Bockmayr M, Samek W, Klauschen F, Müller K-R, Binder A (2020). Resolving challenges in deep learning-based analyses of histopathological images using explanation methods. Sci. Rep..

[11] Wulczyn E, Steiner DF, Moran M, Plass M, Reihs R, Tan F, Flament-Auvigne I, Brown T, Regitnig P, Chen PH, Hegde N (2021). Interpretable survival prediction for colorectal cancer using deep learning. NPJ Digit. Med..

[12] Faust K, Roohi A, Leon AJ, Leroux E, Dent A, Evans AJ, Pugh TJ, Kalimuthu SN, Djuric U, Diamandis P (2020). Unsupervised resolution of histomorphologic heterogeneity in renal cell carcinoma using a brain tumor-educated neural network. JCO Clin. Cancer Inform..

[13] Deng, J. *et al.* Imagenet: a large-scale hierarchical image database. In *2009 IEEE Conference on Computer Vision and Pattern Recognition*, 248–255 (IEEE, 2009).

[14] Ray B, Henaff M, Ma S, Efstathiadis E, Peskin ER, Picone M, Poli T, Aliferis CF, Statnikov A (2014). Information content and analysis methods for multi-modal high-throughput biomedical data. Sci. Rep..

[15] Kong J, Cooper LA, Wang F, Gutman DA, Gao J, Chisolm C, Sharma A, Pan T, Van Meir EG, Kurc TM, Moreno CS (2011). Integrative, multimodal analysis of glioblastoma using TCGA molecular data, pathology images, and clinical outcomes. IEEE Trans. Biomed. Eng..

[16] Schmauch B, Romagnoni A, Pronier E, Saillard C, Maillé P, Calderaro J, Kamoun A, Sefta M, Toldo S, Zaslavskiy M (2020). A deep learning model to predict RNA-Seq expression of tumours from whole slide images. Nat. Commun..

[17] Levy-Jurgenson A, Tekpli X, Kristensen VN, Yakhini Z (2020). Spatial transcriptomics inferred from pathology whole-slide images links tumor heterogeneity to survival in breast and lung cancer. Sci. Rep..

[18] Badea L, Stănescu E (2020). Identifying transcriptomic correlates of histology using deep learning. PLoS ONE.

[19] Rawat RR, Ortega I, Roy P, Sha F, Shibata D, Ruderman D, Agus DB (2020). Deep learned tissue "fingerprints" classify breast cancers by ER/PR/Her2 status from H&E images. Sci. Rep..

[20] Szegedy, C., Vanhoucke, V., Ioffe, S., Shlens, J. & Wojna, Z. Rethinking the inception architecture for computer vision. In *Proceedings of the IEEE Conference on Computer Vision and Pattern Recognition*, 2818–2826 (2016).

[21] Foroughi pour A, Dalton LA (2020). Theory of optimal Bayesian feature filtering. Bayesian Anal..

[22] Ambros RA, Trost R (1990). Cellularity in breast carcinoma. Am. J. Clin. Pathol..

[23] Tanaka K, Yamamoto D, Yamada M, Okugawa H (2004). Influence of cellularity in human breast carcinoma. Breast.

[24] Serrablo A, Paliogiannis P, Pulighe F, Moro SS, Borrego-Estella V, Attene F, Scognamillo F, Hörndler C (2016). Impact of novel histopathological factors on the outcomes of liver surgery for colorectal cancer metastases. Eur. J. Surg. Oncol..

[25] Fliedner FP, Engel TB, El-Ali HH, Hansen AE, Kjaer A (2020). Diffusion weighted magnetic resonance imaging (DW-MRI) as a non-invasive, tissue cellularity marker to monitor cancer treatment response. BMC Cancer.

[26] Park J, Chung YR, Kong ST, Kim YW, Park H, Kim K, Kim DI, Jung KH (2021). Aggregation of cohorts for histopathological diagnosis with deep morphological analysis. Sci. Rep..

[27] Buys TPH, Chari R, Lee EH, Zhang M, MacAulay C, Lam S, Lam WL, Ling V (2007). Genetic changes in the evolution of multidrug resistance for cultured human ovarian cancer cells. Genes Chromosomes Cancer.

[28] Zhang W, Liu Y, Sun N, Wang D, Boyd-Kirkup J, Dou X, Han JD (2013). Integrating genomic, epigenomic, and transcriptomic features reveals modular signatures underlying poor prognosis in ovarian cancer. Cell Rep..

[29] Li S, Li H, Ying X, Lv X (2017). Identification of candidate biomarkers for epithelial ovarian cancer metastasis using microarray data. Oncol. Lett..

[30] Sterzyńska K, Klejewski A, Wojtowicz K, Świerczewska M, Nowacka M, Kaźmierczak D, Andrzejewska M, Rusek D, Brązert M, Brązert J (2019). Mutual expression of ALDH1A1, LOX, and collagens in ovarian cancer cell lines as combined CSCs-and ECM-related models of drug resistance development. Int. J. Mol. Sci..

[31] Rooney MS, Shukla SA, Wu CJ, Getz G, Hacohen N (2015). Molecular and genetic properties of tumors associated with local immune cytolytic activity. Cell.

[32] Cari L, De Rosa F, Petrillo MG, Migliorati G, Nocentini G, Riccardi C (2019). Identification of 15 T cell restricted genes evaluates T cell infiltration of human healthy tissues and cancers and shows prognostic and predictive potential. Int. J. Mol. Sci..

[33] Thorsson V, Gibbs DL, Brown SD, Wolf D, Bortone DS, Yang TH, Porta-Pardo E, Gao GF, Plaisier CL, Eddy JA, Ziv E (2018). The immune landscape of cancer. Immunity.

[34] Geng Z-H, Ye C-X, Huang Y, Jiang H-P, Ye Y-J, Wang S, Zhou Y, Shen Z-L, Qiu X-Y (2019). Human colorectal cancer cells frequently express IGG and display unique IG repertoire. World J. Gastrointest. Oncol..

[35] National Cancer Institute Clinical Proteomic Tumor Analysis Consortium. Radiology data from the clinical proteomic tumor analysis consortium lung adenocarcinoma [cptac-luad] collection [data set]. *Cancer Imaging Archive* (2018).

[36] National Cancer Institute Clinical Proteomic Tumor Analysis Consortium. Radiology data from the clinical proteomic tumor analysis consortium lung squamous cell carcinoma [cptac-lscc] collection [data set]. *Cancer Imaging Archive* (2018).

[37] Ash JT, Darnell G, Munro D, Engelhardt BE (2021). Joint analysis of expression levels and histological images identifies genes associated with tissue morphology. Nat. Commun..

[38] Fu Y, Jung AW, Torne RV, Gonzalez S, Vöhringer H, Shmatko A, Yates LR, Jimenez-Linan M, Moore L, Gerstung M (2020). Pan-cancer computational histopathology reveals mutations, tumor composition and prognosis. Nat. Cancer.

[39] Kather JN, Heij LR, Grabsch HI, Loeffler C, Echle A, Muti HS, Krause J, Niehues JM, Sommer KA, Bankhead P (2020). Pan-cancer image-based detection of clinically actionable genetic alterations. Nat. Cancer.

[40] Ahmad S, Scheinkman L (2019). How can we be so dense? The robustness of highly sparse representations. CoRR.

[41] Rakin, A. S. *et al.* Robust sparse regularization: Simultaneously optimizing neural network robustness and compactness. arXiv preprint arXiv:1905.13074 (2019).

[42] Sun, Y., Xiong, W. & Liang, F. Sparse deep learning: A new framework immune to local traps and miscalibration. *Adv. Neural Inf. Process. Syst.* **34** (2021).

[43] Ternes, L. *et al.* Me-vae: Multi-encoder variational autoencoder for controlling multiple transformational features in single cell image analysis. *bioRxiv* (2021). 10.1038/s42003-022-03218-x PMC8943013 35322205

[44] McNeely-White D, Beveridge JR, Draper BA (2020). Inception and ResNet features are (almost) equivalent. Cogn. Syst. Res..

[45] Jackson CR, Sriharan A, Vaickus LJ (2020). A machine learning algorithm for simulating immunohistochemistry: Development of SOX10 virtual IHC and evaluation on primarily melanocytic neoplasms. Mod. Pathol..

[46] Xu, Z. Moro, C. F., Bozóky, B. & Zhang, Q. Gan-based virtual re-staining: a promising solution for whole slide image analysis. arXiv preprint arXiv:1901.04059 (2019).

[47] Benjamini Y, Hochberg Y (1995). Controlling the false discovery rate: A practical and powerful approach to multiple testing. J. R. Stat. Soc. Ser. B Methodol..

[48] Virtanen P, Gommers R, Oliphant TE, Haberland M, Reddy T, Cournapeau D, Burovski E, Peterson P, Weckesser W, Bright J, Van Der Walt SJ (2020). Scipy 1.0: Fundamental algorithms for scientific computing in python. Nat. Methods.

[49] Seabold, S. & Perktold, J. statsmodels: Econometric and statistical modeling with python. In *9th Python in Science Conference* (2010).

[50] Foroughi pour A, Dalton LA (2019). Bayesian error analysis for feature selection in biomarker discovery. IEEE Access.

[51] Foroughi pour A, Dalton LA (2018). Optimal Bayesian filtering for biomarker discovery: Performance and robustness. IEEE/ACM Trans. Comput. Biol. Bioinform..

[52] Foroughi pour, A. & Dalton, L. A. Biomarker discovery via optimal Bayesian feature filtering for structured multiclass data. In *Proceedings of the 2018 ACM International Conference on Bioinformatics, Computational Biology, and Health Informatics*, 331–340 (2018).

[53] Ledoit O, Wolf M (2004). A well-conditioned estimator for large-dimensional covariance matrices. J. Multivariate Anal..

[54] Pedregosa F, Varoquaux G, Gramfort A, Michel V, Thirion B, Grisel O, Blondel M, Prettenhofer P, Weiss R, Dubourg V, Vanderplas J, Passos A, Cournapeau D, Brucher M, Perrot M, Duchesnay E (2011). Scikit-learn: Machine learning in Python. J. Mach. Learn. Res..

[55] Michael Waskom and the seaborn development team. (Seaborn, 2020).

[56] Defazio, A., Bach, F. & Lacoste-Julien, S. Saga: A fast incremental gradient method with support for non-strongly convex composite objectives. arXiv preprint arXiv:1407.0202 (2014).

[57] Stringer C, Wang T, Michaelos M, Pachitariu M (2021). Cellpose: A generalist algorithm for cellular segmentation. Nat. Methods.

[58] Graham S, Vu QD, Raza SE, Azam A, Tsang YW, Kwak JT, Rajpoot N (2019). Hover-net: Simultaneous segmentation and classification of nuclei in multi-tissue histology images. Med. Image Anal..

[59] Gamper, J. *et al.* Pannuke dataset extension, insights and baselines. arXiv preprint arXiv:2003.10778 (2020).

[60] Grossman RL, Heath AP, Ferretti V, Varmus HE, Lowy DR, Kibbe WA, Staudt LM (2016). Toward a shared vision for cancer genomic data. N. Engl. J. Med..

[61] Clark K, Vendt B, Smith K, Freymann J, Kirby J, Koppel P, Moore S, Phillips S, Maffitt D, Pringle M (2013). The cancer imaging archive (TCIA): Maintaining and operating a public information repository. J. Digit. Imaging.

[62] Guo, M-H. *et al.* Attention mechanisms in computer vision: A survey. arXiv preprint arXiv:2111.07624 (2021).

