# Supplementary material for: Deep learning features encode interpretable morphologies within histological images
Source: Sci Rep. 2022 Jun 8;12:9428. doi: 10.1038/s41598-022-13541-2 (PMC9177767; doi:10.1038/s41598-022-13541-2)
Supplement: Supplementary file 2 — Supplementary Information. [file 41598_2022_13541_MOESM2_ESM.zip › Sfiles/Sfile3.pdf]

Submitted to the Annals of Probability
0000 Vol. 00, No. 0, 1
DOI: 0000

PAN-CANCER AND CANCER SPECIFIC MONES WITHIN EACH CANCER FAMILY

1

TABLE 1
Expectation, standard deviation, and number of significant pan-cancer and cancer-specific mones among kidney cancers only using frozen slides (Y/N: mones have/do not have distributional difference between tumor and normal slides of cancer).

KICH	KIRC	KIRP	Expectation	std.	5% FDR-OBF
Y	Y	Y	1157	9	1146
Y	Y	N	298	8	193
Y	N	Y	78	4	51
N	Y	Y	246	8	137
Y	N	N	39	3	17
N	Y	N	169	7	72
N	N	Y	30	3	9

TABLE 2
Expectation, standard deviation, and number of significant pan-cancer and cancer-specific mones among pan-GYN cancers only using frozen slides (Y/N: mones have/do not have distributional difference between tumor and normal slides of cancer).

BRCA	OV	UCEC	Expectation	std.	5% FDR-OBF
Y	Y	Y	1384	9	1415
Y	Y	N	229	7	164
Y	N	Y	231	6	178
N	Y	Y	99	4	84
Y	N	N	69	4	35
N	Y	N	14	2	7
N	N	Y	17	2	10

TABLE 3
Expectation, standard deviation, and number of significant pan-cancer and cancer-specific mones among pan-GI cancers only using frozen slides (Y/N: mones have/do not have distributional difference between tumor and normal slides of cancer).

COAD	READ	STAD	Expectation	std.	5% FDR-OBF
Y	Y	Y	888	10	829
Y	Y	N	241	7	172
Y	N	Y	273	10	85
N	Y	Y	129	7	32
Y	N	N	125	7	23
N	Y	N	54	5	13
N	N	Y	243	8	94

TABLE 4

Expectation, standard deviation, and number of significant pan-cancer and cancer-specific mones among lung cancers only using frozen slides (Y/N: mones have/do not have distributional difference between tumor and normal slides of cancer).

LUAD	LUSC	tumor subtype	Expectation	std.	5% FDR-OBF
Y	Y	Y	990	9	988
Y	Y	N	488	8	448
Y	N	Y	97	5	61
N	Y	Y	186	6	134
Y	N	N	54	4	28
N	Y	N	88	5	51
N	N	Y	93	4	63

TABLE 5

Expectation, standard deviation, and number of significant pan-cancer and cancer-specific mones among lung cancers only using frozen slides (Y/N: mones have/do not have distributional difference between tumor and normal slides of cancer).

LUAD	LUSC	tissue of origin	Expectation	std.	5% FDR
Y	Y	Y	549	9	469
Y	Y	N	929	10	872
Y	N	Y	63	4	35
N	Y	Y	103	5	62
Y	N	N	88	5	45
N	Y	N	171	6	95
N	N	Y	48	4	26
