# Supplementary material for: Deep learning features encode interpretable morphologies within histological images
Source: Sci Rep. 2022 Jun 8;12:9428. doi: 10.1038/s41598-022-13541-2 (PMC9177767; doi:10.1038/s41598-022-13541-2)
Supplement: Supplementary file 2 — Supplementary Information. [file 41598_2022_13541_MOESM2_ESM.zip › Sfiles/Sfile4.docx]

**Mones patterns can be affected by data type**

Mones differentiate between frozen and FFPE tumor slides (see methods, see the Figure below). Many of the mones distinguishing frozen adjacent normal and froze tumors showed stronger distributional differences between frozen tumor and adjacent normal slides than between frozen tumor and FFPE tumor (606±132,see Supplementary File 12). Several hundred mones (421±112) are strongly affected by differences between data modalities (see Supplementary File 12). Liver hepatocellular carcinoma (LIHC) had the largest number of such mones (593 mones) and OV had the lowest (213 mones). Interestingly, 590±163 mones had more similarities between frozen adjacent normals and FFPE tumor slides than across tumor slides (FFPE and frozen)(see methods, see Supplementary File 12). These mones may have lost the ability to measure their respective morphological features due to differences between frozen and FFPE slides.


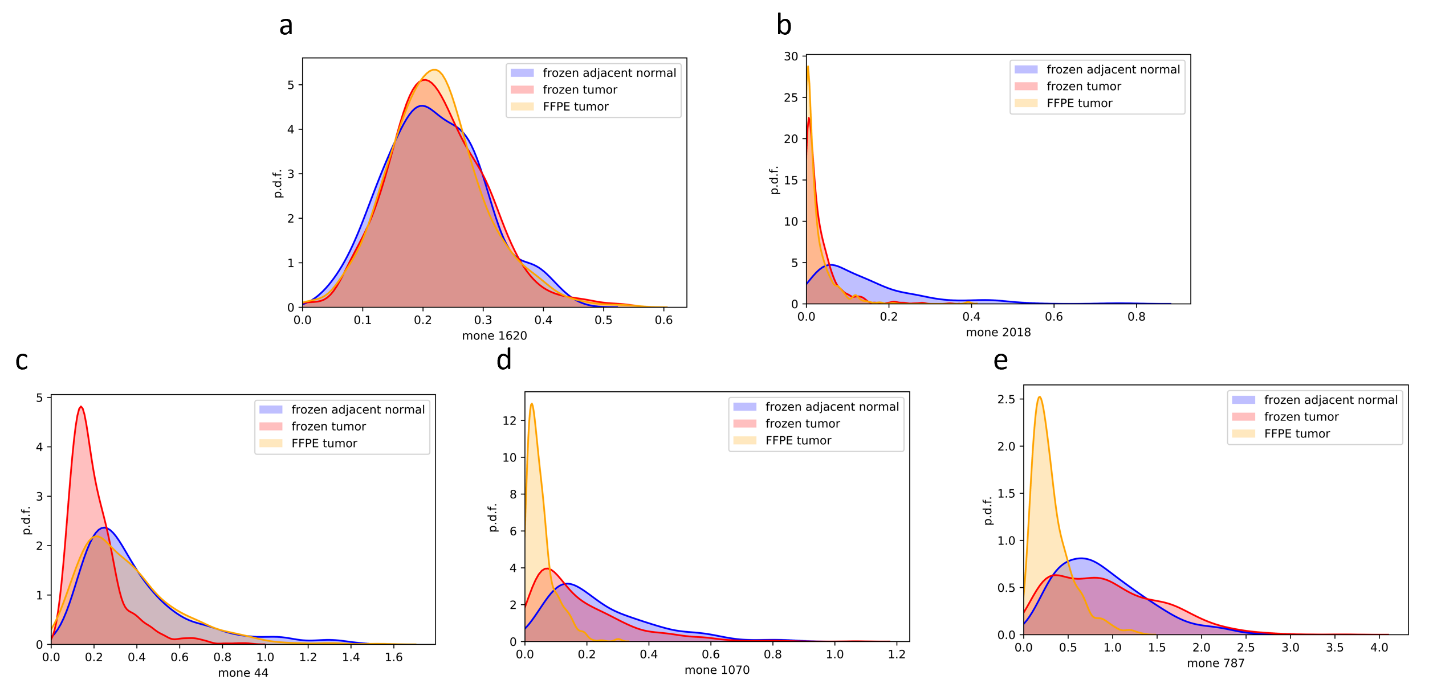


**Figure:** Distribution of various LUAD mones across frozen tumor, frozen normal, and FFPE slides: (a) a non-marker mone not separating any of the classes (structure A), (b) a mone separating tumor and normal slides with similar distribution across FFPE and frozen tumor slides (structure C), (c) a mone with similar distributions among frozen normal and FFPE slides (structure D), (d,e) A mone with more similar distributions across frozen slides than FFPE (Structure B). (d) Both FFPE and frozen tumor slides have smaller medians than frozen normals. (e) Frozen tumors have higher medians than frozen normals but FFPE slides have smaller medians. See the methods section on structured multiclass OBF for the descriptions of the different mone structures.
